# Supplementary material for: Three-dimensional multi-site random access photostimulation (3D-MAP)
Source: eLife. 2022 Feb 14;11:e73266. doi: 10.7554/eLife.73266 (PMC8843094; doi:10.7554/eLife.73266)
Supplement: Transparent reporting form [file elife-73266-transrepform1.pdf]

eLIFE
1st Floor
24 Hills Road
Cambridge CB2 1JP, UK
P 01223 855340
W elifesciences.org
T @elife

eLife's transparent reporting form

We encourage authors to provide detailed information *within their submission* to facilitate the interpretation and replication of experiments. Authors can upload supporting documentation to indicate the use of appropriate reporting guidelines for health-related research (see EQUATOR Network), life science research (see the BioSharing Information Resource), or the ARRIVE guidelines for reporting work involving animal research. Where applicable, authors should refer to any relevant reporting standards documents in this form.

If you have any questions, please consult our Journal Policies and/or contact us: editorial@elifesciences.org.

Sample-size estimation

- You should state whether an appropriate sample size was computed when the study was being designed
- You should state the statistical method of sample size computation and any required assumptions
- If no explicit power analysis was used, you should describe how you decided what sample (replicate) size (number) to use

Please outline where this information can be found within the submission (e.g., sections or figure legends), or explain why this information doesn't apply to your submission:

A priori sample size estimation was not performed for all individual experiments. However, from experience measurements from whole cell recordings require at least 5-10 observations (light response in a specific location) to resolve a difference of ~25%, as typical observed differences covered an order of magnitude these 5-10 observations were more than sufficient for our needs. Furthermore, within a cell there was very little trial to trial variability so only 3-5 repetitions of a given stimulus were needed for an accurate report of the optical response at a given location. In the all-optical experiments, the calcium traces are averaged across 10 repetitions. All-optical PPSF is averaged across 10 neurons.

Replicates

- You should report how often each experiment was performed
- You should include a definition of biological versus technical replication
- The data obtained should be provided and sufficient information should be provided to indicate the number of independent biological and/or technical replicates
- If you encountered any outliers, you should describe how these were handled
- Criteria for exclusion/inclusion of data should be clearly stated
- High-throughput sequence data should be uploaded before submission, with a private link for reviewers provided (these are available from both GEO and ArrayExpress)

Please outline where this information can be found within the submission (e.g., sections or figure legends), or explain why this information doesn't apply to your submission:

eLife Sciences Publications, Ltd is a limited liability non-profit non-stock corporation incorporated in the State of Delaware, USA, with company number 5030732, and is registered in the UK with company number FC030576 and branch number BR015634 at the address 1st Floor, 24 Hills Road, Cambridge CB2 1JP | August 2014

Repeats of all electrophysiology experiments were made across >3 mice of identical strain and expression condition. Repeats of all-optical experiments were made across 2-4 mice of identical strain and expression condition. All biological replicates were successful. We also performed repeat measurements for all the experiments in the manuscript. The number of the technical replication is stated with each experiment in the manuscript.

Patch clamp electrophysiological recordings were excluded when the experiment did not last long enough to obtain >3 samples per location. Experiments were terminated when the cell holding current at -60 mV exceeded 500 pA or the series resistance increased by more than 25% from the beginning of data acquisition. In animals in which viral opsin expression was insufficient to spike neurons (a small minority of cases) data from those animals was excluded. In the all-optical experiments, neurons in the field-of-view but do not co-express opsin and calcium indicators, or co-expressed neurons but no calcium activity is detected under all conditions are excluded.

Statistical reporting

- Statistical analysis methods should be described and justified
- Raw data should be presented in figures whenever informative to do so (typically when N per group is less than 10)
- For each experiment, you should identify the statistical tests used, exact values of N, definitions of center, methods of multiple test correction, and dispersion and precision measures (e.g., mean, median, SD, SEM, confidence intervals; and, for the major substantive results, a measure of effect size (e.g., Pearson's r, Cohen's d)
- Report exact p-values wherever possible alongside the summary statistics and 95% confidence intervals. These should be reported for all key questions and not only when the p-value is less than 0.05.

Please outline where this information can be found within the submission (e.g., sections or figure legends), or explain why this information doesn't apply to your submission:

In the measurement of physiological PPSF (Fig. 3) and all-optical PPSF (Fig. 6), we presented the value of the PPSF with mean and SEM.

(For large datasets, or papers with a very large number of statistical tests, you may upload a single table file with tests, Ns, etc., with reference to sections in the manuscript.)

Group allocation

- Indicate how samples were allocated into experimental groups (in the case of clinical studies, please specify allocation to treatment method); if randomization was used, please also state if restricted randomization was applied
- Indicate if masking was used during group allocation, data collection and/or data analysis

Please outline where this information can be found within the submission (e.g., sections or figure legends), or explain why this information doesn't apply to your submission:

Group allocation is not relevant.

Additional data files ("source data")

- We encourage you to upload relevant additional data files, such as numerical data that are represented as a graph in a figure, or as a summary table
- Where provided, these should be in the most useful format, and they can be uploaded as "Source data" files linked to a main figure or table
- Include model definition files including the full list of parameters used
- Include code used for data analysis (e.g., R, MatLab)
- Avoid stating that data files are "available upon request"

Please indicate the figures or tables for which source data files have been provided:

Custom code used to collect and process data is programed in MATLAB. The code has been deposited in Github (https://github.com/Waller-Lab/3D-MAP). Raw data of electrophysiology measurement and optical imaging has been deposited in Dryad (doi:10.6078/D1571M).

eLife Sciences Publications, Ltd is a limited liability non-profit non-stock corporation incorporated in the State of Delaware, USA, with company number 5030732, and is registered in the UK with company number FC030576 and branch number BR015634 at the address 1st Floor, 24 Hills Road, Cambridge CB2 1JP | August 2014
